# HIV infected CD4+ T cell clones are more stable than uninfected clones during long-term antiretroviral therapy

**DOI:** 10.1371/journal.ppat.1010726

**Published:** 2022-08-31

**Authors:** Shuang Guo, Brian T. Luke, Amy R. Henry, Samuel Darko, Leah D. Brandt, Ling Su, David Sun, Daria Wells, Kevin W. Joseph, Dimiter Demirov, Elias K. Halvas, Daniel C. Douek, Xiaolin Wu, John W. Mellors, Stephen H. Hughes

**Affiliations:** 1 Leidos Biomedical Research, Inc., Frederick National Laboratory for Cancer Research, Frederick, Maryland, United States of America; 2 Vaccine Research Center, National Institute of Allergy and Infectious Diseases, Bethesda Maryland, United States of America; 3 Division of Infectious Diseases, Department of Medicine, University of Pittsburgh, Pittsburgh, Pennsylvania, United States of America; 4 HIV Dynamics and Replication Program, CCR, National Cancer Institute, Frederick, Maryland, United States of America; University of Pennsylvania, UNITED STATES

## Abstract

Although combination antiretroviral therapy (ART) blocks HIV replication, it is not curative because infected CD4+ T cells that carry intact, infectious proviruses persist. Understanding the behavior of clones of infected T cells is important for understanding the stability of the reservoir; however, the stabilities of clones of infected T cells in persons on long-term ART are not well defined. We determined the relative stabilities of clones of infected and uninfected CD4+ T cells over time intervals of one to four years in three individuals who had been on ART for 9–19 years. The largest clones of uninfected T cells were larger than the largest clones of infected T cells. Clones of infected CD4+ T cells were more stable than clones of uninfected CD4+ T cells of a similar size. Individual clones of CD4+ T cells carrying intact, infectious proviruses can expand, contract, or remain stable over time.

## Author summary

In HIV infected individuals, infected T cells can clonally expand and persist for many years, which is one of the primary reasons current anti-retroviral therapy (ART), which blocks viral replication, does not cure HIV infections. We compared the relative stabilities of clones of infected and uninfected T cells in three donors who had been on successful long-term ART for more than 9 years. The clones of infected T cells were much more stable than similar-sized clones of uninfected T cells. We were initially surprised by this result but, because ART blocks viral replication, we realized that all of the infected clones must be “old” and must have originated before ART was initiated. In contrast, the clones of uninfected cells would have included both old and new clones. In addition, we looked at the behavior of three clones (one in each donor) each of which carries an intact, replication-competent, provirus. The data show that even after more than 9 years, clones that carry replication-competent proviruses can either increase or decrease in size.

## Introduction

Combination antiretroviral therapy (ART) effectively blocks HIV replication but does not cure HIV infection because DNA copies of HIV genomes are integrated into the host cell genome, forming proviruses. Proviruses persist in HIV infected individuals on ART for as long as the infected host cells, or their progeny, survive. HIV preferentially infects CD4+ T cells. Infected CD4+ T cells can clonally expand and clones of infected cells can persist for more than ten years [1,2]. The original infected T cell, and all of its progeny, carry the same provirus integrated in exactly the same place in the human genome. Similarly, all of the progeny of an uninfected or an HIV infected T cell have the identical T cell receptor.

In persons living with HIV who are on effective ART, most (95–98%) of the infected CD4+ T cells carry defective copies of the HIV genome [3]; however, a small fraction of the clones carry intact, infectious HIV proviruses [4, 5]. The cells that carry intact, infectious proviruses form the reservoir that has made it impossible to achieve an HIV cure with any of the current ART regimens. The reservoir is formed, and clones of infected cells start to expand, shortly after an individual is infected [6, 7] [8]. Large clones of infected cells (>10^5^ cells) can be found a few weeks after a person acquires an HIV infection. Some of the clones of infected cells that arise in the first few weeks after the initial infection persist for years on ART [8, 9].

In those on ART, a small fraction (2.2%) of the infected CD4+ T cells that have clonally expanded have a provirus integrated in one of seven oncogenes: *BACH2*, *STAT5B*, *MKL2*, *MKL1*, *IL2RB*, *MYB*, and *POU2F1* [10]. Analysis of the data strongly suggests that proviruses integrated in particular introns of these oncogenes, in the same transcriptional orientation as the oncogene, can drive the expression of the oncogene, which enhances the growth of the cells and/or their survival [10]. These findings suggest that, although integrations in oncogenes can cause the clonal expansion of HIV infected cells in vivo, the oncogene-driven clones make only a minor contribution to the overall clonal expansion of infected T cells that occurs both before and during ART. Although more work is needed, it appears that the majority of the infected cells that have clonally expanded have done so in response to signals that lead to the clonal expansion of uninfected T cells, i.e. antigen and cytokine stimulation [10–12].

In the absence of ART, only a small fraction of the T cells that are infected go on to form clones [10]. Viral proteins are toxic and the host’s immune response can kill cells that are producing viral proteins. Although the available data are limited, it appears that only a small fraction (<5%) of the cells in infected clones in those on ART express viral RNA, and by extension, viral proteins, at any one time [13]; however, it was recently reported that a higher fraction of infected cell clones can express viral RNA [14]. This appears to be true both for clones that carry defective and intact proviruses and there are data showing that many defective proviruses can still express viral antigens [15–17]. These findings suggest that there could be differences in the properties and behavior of clones of infected vs. uninfected T cells. We initially thought that clones of infected T cells might be less stable than clones of uninfected T cells.

To explore the possibility that clones of HIV-infected and uninfected CD4+ T cells behave differently, we obtained billions of purified CD4+ T cells from three individuals on ART and compared the sizes, and the stabilities over periods of time from one to four years, of clones of infected and uninfected cells from each of the donors. We used integration site analysis to identify clones of infected cells and to measure their relative sizes. Given that most CD4+ T cells are uninfected (>99%), we were able to estimate the sizes of clones of uninfected CD4+ T cells by analyzing the sequences of the T cell receptors (TCRs) from the same samples that were used for the integration site analysis [18]. Because we were interested in the behavior of the large clones of infected and uninfected cells, the TCR analysis was done with populations of CD4+ T cells from which the naïve T cells had been removed. However, despite our initial expectations, a comparison of the sizes of individual clones of infected and uninfected of CD4+ T cells over periods of from one to four years showed that the sizes of clones of infected CD4+ T cells were, on average, more stable than were clones of uninfected CD4+ T cells in samples from identical time points (see Discussion).

## Results

### Selection of donors

We studied HIV-positive donors on ART who, despite therapeutic drug levels and no evidence of drug resistance, had persistent levels of HIV-1 RNA in the plasma above the limit of detection [5]. We chose to analyze samples from these three donors because they provided an opportunity to look broadly at the stability of large populations of infected and uninfected cell clones and allowed us to measure the stability of clones of infected cells that carry intact infectious proviruses. The characteristics of the three donors who provided the samples are shown in Table 1. We identified, in each of the three donors, a large clone of infected cells that carried an intact, infectious provirus. In two of the 3 donors (C-03 and R-09) the non-suppressible viremia was shown to be the result of virus that was produced by the large clone of cells that carried the infectious provirus that we identified. Thus, in these donors, most of the clones that carry an intact, infectious provirus behave like clones that carry intact, infectious proviruses in donors whose viral load is undetectable: they produce little or no virus. Longitudinal genetic analysis of viremia by single genome sequencing showed no evidence of evolution from ongoing viral replication [5]. In the third donor (F-07), the sequence of the provirus in the large clone carrying an intact infectious provirus did not match the sequences of the virus in the blood. The virus in the blood appeared to be produced by clones of infected cells that we did not identify in the blood samples. All three donors were males and they ranged in age from 43 to 73 (Table 1). All had been on ART for between 9 and 19 years and had clinically detectable clonal viremia for between 2.1 and 5.2 years. The oldest donor, R-09, was diagnosed with Hodgkin’s lymphoma in January of 2008. He was treated with adriamycin, bleomycin, vinblastine, and dacarbazine, with the last treatment given in June 2009, approximately 6 years before the first sample was taken for the current study.

**Table 1:** Demographics and Clinical Characteristics of Donors with Non-Suppressible Viremia on Antiretroviral Therapy (ART).

Donor	Age	Sex	Race	Date of HIV Diagnosis	Years on ART^a^	Years of Non-Suppressible Viremia^b^
R-09	73	Male	Caucasian	01/2005	10	5.2
C-03	43	Male	Caucasian	06/1994	9	4.5
F-07	59	Male	African American	06/1996	19	2.1

^a^ Years on ART at the time of initial evaluation

^b^ Above the limit of detection of FDA-approved HIV-1 RNA assays; sequence analysis of viral RNA in plasma showed clonal or oligoclonal viremia that did not evolve longitudinally [5]

### Integration site analysis of PBMCs and CD4+ T cells to characterize the clones of infected cells

To obtain adequate sampling of the distribution of the sizes of clones of infected cells, we identified more than 19,000 integration sites from each of the three donors [Table 2; the integration site data can be found in the HIV-DRP Retroviral Integration Site Database (RID)]. Integration site analysis was performed on genomic DNA extracted from cells taken at two or three time points from each of the donors (Table 2). We compared the sizes of clones of infected cells at different time points to determine whether the sizes of the clones changed over time. Despite the large numbers of integration sites obtained, analysis was restricted to the largest 100 clones of infected cells because the sizes of smaller clones meant that the size measurements were subject to sampling errors (further explanation is provided in the S1 Text). We determined, in parallel, the sizes of the clones of all of the CD4+ T cells from the corresponding time points from the same donors by analyzing the RNA sequences from the expressed T cell receptors (TCRs) [18] in CD4+ cells from which the naïve cells had been removed. The naïve cells were removed because we were interested in the behavior of the large clones; naïve cells have not been stimulated by their cognate antigens and, for that reason, there are no large clones of naïve cells. Most of the CD4+ T cells (> 99%) were uninfected, which allowed us to use the sizes of all the clones (determined by TCR analysis) as a measure of the sizes of uninfected T cell clones.

**Table 2:** Integration Sites Obtained from the Three Donors.

Donor	Date	Cell Type	Integration Sites	Total Integration Sites for Each Date	Total Integration Sites for Each Donor
R-09	8/6/2015	PBMC	118	5,530	19,230
		CD4	5,412		
	9/14/2016	PBMC	4,316	13,700	
		CD4	9,384		
C-03	9/30/2014	CD4	2,077	2,077	19,712
	11/30/2015	PBMC	751	8,695	
		CD4	7,944		
	10/5/2017	CD4	8,940	8,940	
F-07	6/3/2015	PBMC	944	2,516	20,185
		CD4	1,572		
	4/6/2017	CD4	13,226	13,226	
	4/3/2019	CD4	4,443	4,443	

We began by asking if all of the large clones of infected cells that were identified in PBMC samples from one of the donors (R-09) were also detected in negatively selected CD4+ T cells (see Materials and Methods). Integration sites are more easily and efficiently recovered from CD4+ T cells than from PBMC, but to ensure that by analyzing purified CD4+ T cells we did not overlook any large clones, we also analyzed PBMCs from the same time points. Using the data in S1 Table we showed that each of the 100 largest clones identified from PBMCs was also detected in CD4+ T cells. This analysis shows that the largest clones of HIV-infected cells in blood could be detected using purified CD4+ T cells for the integration site analysis and the majority of the integration site analyses was done using CD4+ T cells (Table 2).

### Sizes and stabilities of large clones of infected T cells

Large integration site datasets provide a much more accurate measure of the sizes of the clones and make it possible to detect smaller clones that would have been missed if a smaller dataset was used (see S1 Text). Using the large integration site datasets, we found that from 43% to 58% of the integration sites were in clones of expanded cells. As expected, we identified large numbers of small clones of infected cells that were not detected when smaller datasets were analyzed [1, 2] (Table 3). Making the assumption that the clones are well-partitioned between the tissues and the blood, we would expect to be able to identify, using the large integration site datasets presented here, clones larger than about 10^4^ cells, approximately an order of magnitude smaller than the smallest clones of infected cells that could have been detected in previous analyses [8].

**Table 3:** Integration Sites Recovered and Numbers of Infected Clones.

Donor	Date Samples Taken	Total Integration Sites	Unique Integration Sites	Number of Clones	Number of Integration Sites in Clones	Percent of Integration Sites in Clones
R-09	8/6/2015	5530	3536	1209	3121	56.4
R-09	9/14/2016	13700	7192	1689	8076	58.9
F-07	6/3/2015	2516	1764	524	1231	48.9
F-07	4/6/2017	13226	8605	1186	5415	40.9
F-07	4/3/2019	4443	3013	745	2101	47.3
C-03	9/30/2014	2077	1611	559	995	47.9
C-03	11/30/2015	8695	6147	1352	3660	42.1
C-03	10/5/2017	8940	5841	1305	4201	47.0
C-03	All Dates	19712	12033	1653	8856	44.9
F-07	All Dates	20185	12225	1301	8747	43.3
R-09	All Dates	19230	9596	1768	11197	58.2

The number of unique integration sites is the total number of different integration sites that were found, whether they were, or were not, seen more than once. The total number of integration sites is the number of independent integration sites recovered, which includes a count of the integration sites that were recovered more than once (those in clones). The number of clones is the number of independent integration sites seen at least twice.

Donor R-09 had the largest fraction of integration sites in clones (Table 3). As noted earlier, this donor had been treated for lymphoma. It is possible that the lymphoma and/or the chemotherapy used to treat the lymphoma, combined with the age of the donor (age 73), could have reduced the overall number of CD4+ T cell clones, which could also have affected the number and relative sizes of the infected clones. However, we cannot determine whether any of integration sites we detected only once were from cells that were, or were not, part of a small clone (see Discussion).

We can determine the number of times we isolate an integration site based on the breakpoints in the human DNA that is appended to the ends of the proviruses; the breakpoint data are supplemented by adding random sequences tags to the amplified fragments [Materials and Methods and [1, 19]]. Because we have reported the CD4+ T cell counts for each donor [5], and have measured the fraction of the CD4+ T cells that are infected by qPCR [20], we can use the fraction of the infected T cells in the individual clones to estimate the sizes of the clones of infected T cells. Because all the donors had been on successful ART for a minimum of 9 years, their CD4+ cell counts and the fraction of their CD4+ T cells that were infected were relatively stable. In the experiemts we report here, we used the CD4+ cell count and the fraction of CD4+ cells that were infected at the second time point for each of the donors. The fractional size of a clone of infected or uninfected cells (fraction of the infected or uninfected CD4+ cells represented by the clone) is independent of the sampling as long as the sampling is deep. In making the calculation of the sizes of the infected and uninfected clones we assumed that the cells in the clones are well-distributed between blood and tissues. There are published data which support this assumption in humans [20–22] and in SIV infected macaques [23]. Although it has been reported by others that they did not find strong evidence that infected clones were widely distributed in the blood and tissues of HIV infected individuals, the authors stated that their conclusion was limited by small sample size [24]. We also assumed that about 97–98% of the cells were in tissues. Importantly, the same factors were used in the calculations of the sizes of both the infected and uninfected clones of cells (described below) and, for that reason, the factors we used to calculate the sizes of the clones will not affect on our estimates of the relative stabilities of the infected and uninfected clones of CD4+ T cells.

**Fig 1:**
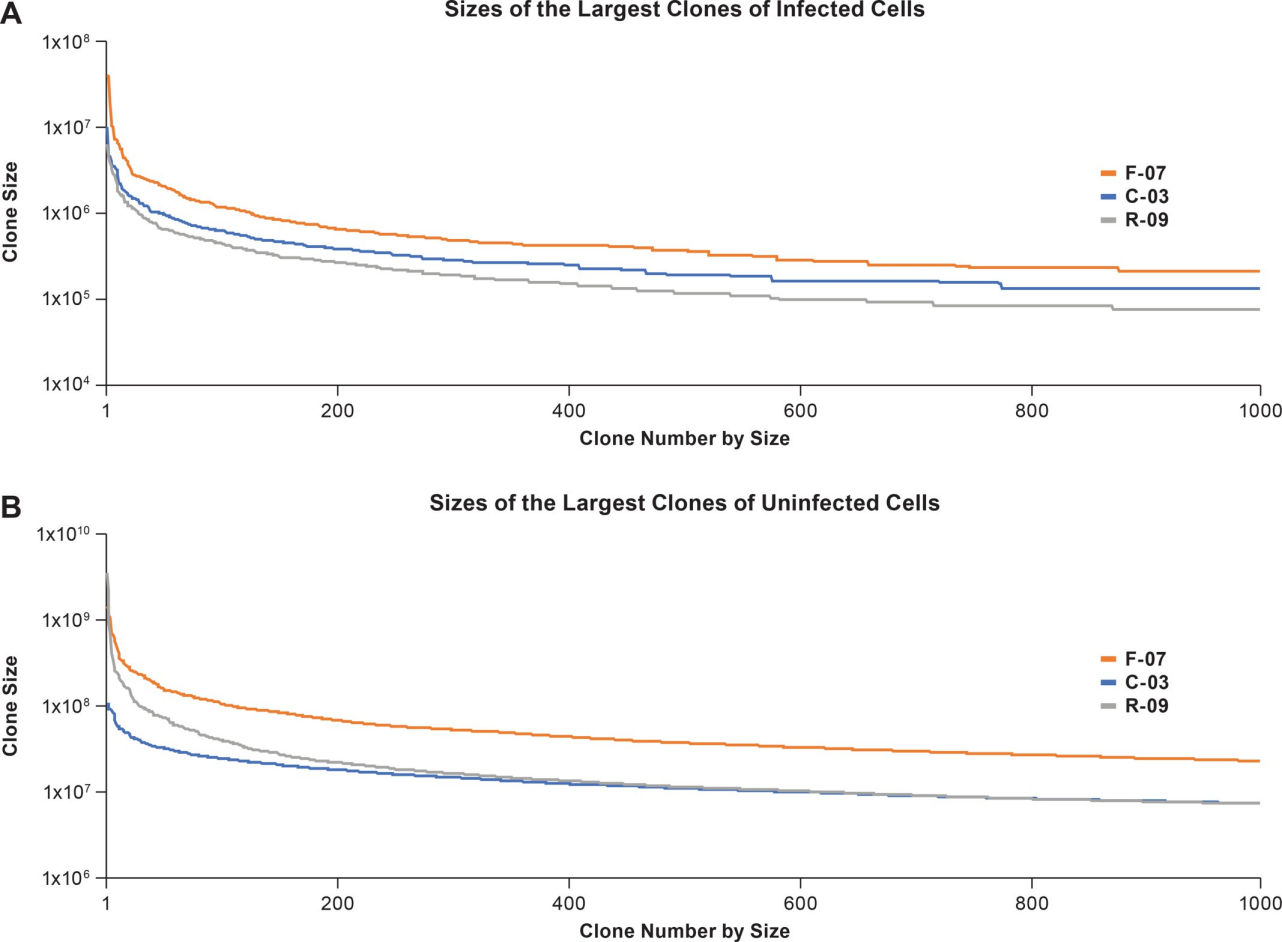
Sizes of the largest clones of infected and uninfected clones of CD4+ T cells in the three donors. The sizes of the largest clones of infected cells were determined using the integration site data (available on the HIV DRP RID). The actual sizes of the individual clones were calculated based on the counts of CD4+ T cells for each of the donors, the fraction of the CD4+ T cells that were infected, and the fraction of the infected cells in each clone. Similarly, the sizes of the uninfected clones were calculated based on the number of CD4+ T cells, and the fraction of the total recovered TCR sequences that were obtained for each clone (see Methods). The clone sizes shown in the figures were determined by combining, for each of the donors, the data obtained from all the time points.

We compared the distributions of the sizes of the largest clones of infected CD4+ T cells from the three donors (Fig 1); the sizes of the largest infected T cell clones were similar. However, the largest infected clones in donor F-07 were larger than the infected clones in the other two donors (see Fig 1), and the largest infected clones in the youngest donor (C-03) were intermediate in size between the clones in F-07 and R-09 (which were, generally speaking, the smallest). Although there may be some small differences in curves, the overall distributions of the sizes of the infected clones were similar for the three donors. We obtained large enough integration site datasets from each of the donors to make meaningful comparisons of the stability, over time, of the largest clones of infected cells (S1 Table, S1 Fig). The data show that there is no obvious correlation between the average sizes of the clones and their tendency to either grow larger, or smaller, over time.

### Size distribution and stability of large clones of uninfected T cells

As mentioned earlier, because only a small fraction (less than 1%) of the CD4+ T cells were HIV infected, TCR analysis can be used to determine the sizes and stabilities of all the clones of CD4+ cells, and the sizes of all the clones can be used as a measure of the sizes and stabilities of the clones of uninfected CD4+ T cells. Put another way, for clones of a similar size, the fraction of the cells that are infected in the donor can be used to predict the fraction of the clones that are infected. Thus, if approximately one cell in 1000 is infected, then, for clones of a similar size, about one clone in 1000 will be infected, which will not significantly affect the analysis. Accordingly, the data for all the clones of CD4+ T cells will be referred to as “clones of uninfected cells”. For the uninfected cells, calculating the sizes of the clones was based on the numbers of TCR sequences obtained in an analysis of CD4+ T cells (from which the naïve cells had been removed) and the overall number of CD4+ T cells in the donor. We have, based on the TCR data, calculated the fraction of the total CD4+ T cells represented by each of the clones. The calculation was similar to the calculation done for the sizes of the clones of infected CD4+ T cells (described above). We used the CD4+ cell count for each donor [5] and we assumed that the cells in the clones of infected and uninfected cells are well-distributed between blood and tissues, and that about 97–98% of the cells were in tissues [21, 25].

We purified CD4+ T cells from the same donors at the same time points that were used to measure the sizes of the clones of infected CD4+ T cells and removed the naïve T cells. Naïve T cells have rearranged T cell receptor genes but have not clonally expanded in response to their cognate antigen. Removing naïve T cells simplifies the TCR analysis that was used to determine the sizes of the clones of CD4+ T cells (Materials and Methods). Because naïve cells have not responded to antigen, they will not have formed large clones, and removing them from the cells we used for the TCR analysis did not affect our ability to detect the large clones of uninfected cells, or determine their sizes. The two largest clones of uninfected CD4+ T cells were obtained from donor R-09, who was both the oldest, and had undergone chemotherapy. When we compared the sizes of the largest 1000 clones, the uninfected clones in donor F-07 were, in general, larger than the clones in donor C-03 or donor R-09 (Fig 1; note that the scales are different in the panels showing the sizes of the clones of uinfected and infected cells). The distribution of the sizes of the uninfected clones in R-09 appears to fall of more rapidly towards smaller sized clones compared to the other donors. However, for uninfected clones smaller than the top 300, the distribution of the sizes of the clones in all the donors is quite similar; this result is modestly different from the result observed with clones of infected cells (Fig 1) (see Discussion).

All the donors had a number of very large clones of uninfected cells (>10^8^); 5 of the 6 of the largest clones of infected cells (>10^7^) were found in donor F-07; the largest was 4.04 X10^7^. C-03 had one infected clone of 1.0X10^7^ cells, the largest clone in R-09 was 6.3 X10^6^. None of the largest clones of infected cells were as large as the largest clones of uninfected cells. There were clones of uninfected CD4+ T cells that were >10^9^ in R-09 and F-07; the largest, in R-09, was 3.5X10^9^. The two largest clones of uninfected cells in C-03 were slightly larger than 10^8^ cells; however, these clones of uninfected cells were an order of magnitude larger than the largest clones of infected cells from this donor. We compared the sizes of the largest clones of uninfected cells over time (S1 Fig). The largest clones of uninfected cells were more stable over the time periods of our analysis than were the clones of uninfected cells whose sizes were similar to the largest clones of infected cells (approximately 10^6^ to 10^8^ cells). Although, as discussed in the next section, that approach makes it possible to understand the behavior of the individual clones of infected cells over time, it does not provide a simple way to compare the overall changes in the sizes of a group of clones over time.

### Determining the overall stabilities of clones of infected and uninfected CD4+ T cells over time

**Fig 2:**
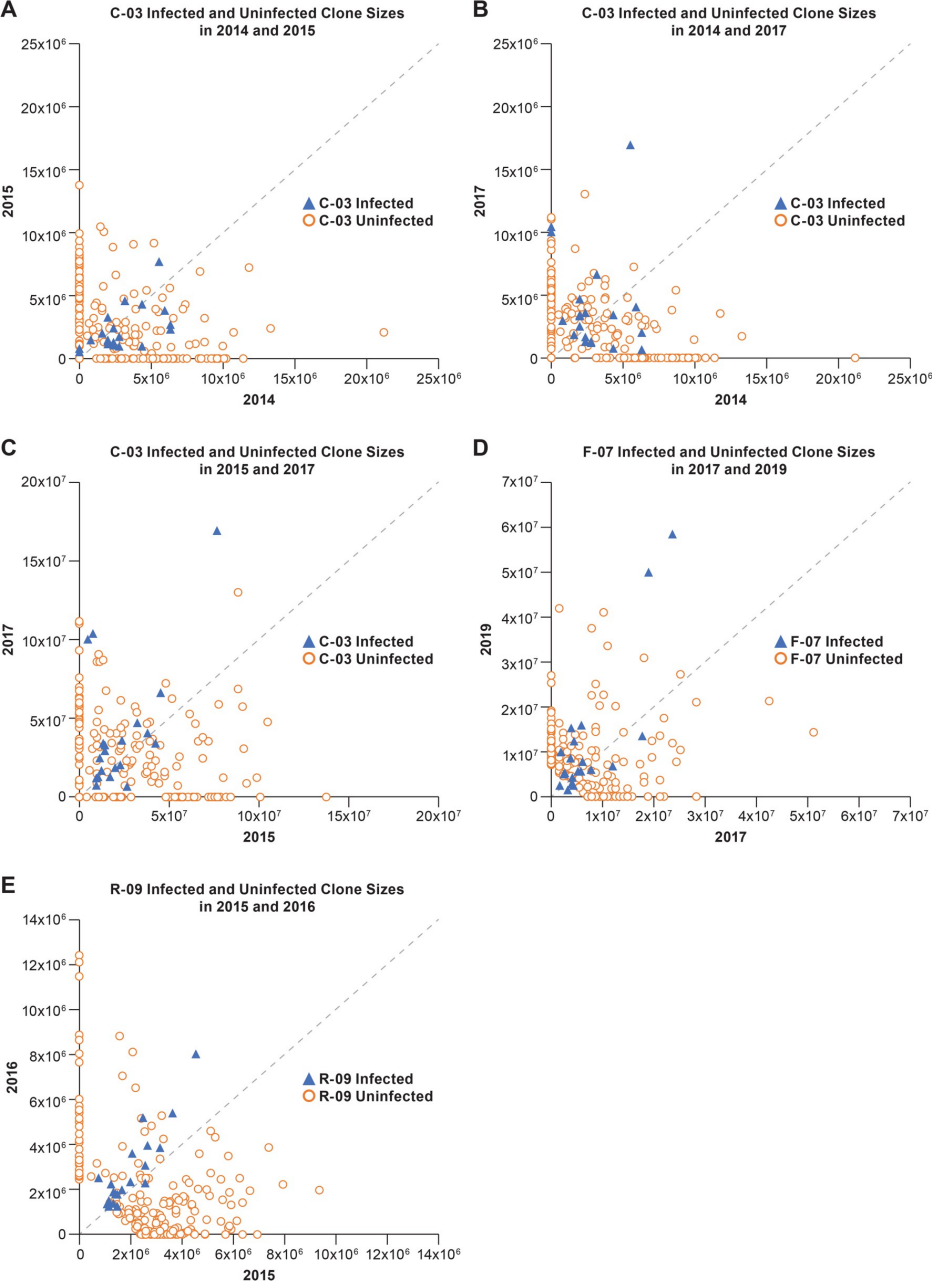
Scatter plots showing the stabilities of clones of infected and uninfected CD4+ T cells. Each panel shows the sizes of the 20 largest clones of infected T cells (blue triangles) and the sizes of 200 clones of uninfected T cells of a similar size at two different times. The dotted line is the diagonal. Any clone whose size does not change between the time points shown on the axes would fall on this diagonal. Similarly, the change in the sizes of each of the clones can be measured by the distance from the diagonal. Each panel **(A-E)** shows a comparison for two time points; the donors and the times are noted in each of the panels.

As noted in the previous paragraph, there is considerable variation in the stabilities of the individual clones, which makes it difficult to compare the relative overall/collective behavior of the largest clones of infected cells in two temporally distinct samples. To better define the variation in the sizes of the clones, we generated scatter plots in which the sizes of the clones of infected and uninfected cells for one time point were plotted on the X axis and for the second time point on the Y axis (Fig 2). The versions of the plots shown in Fig 2 show only the 20 largest clones of infected cells; these are compared to 200 clones of uninfected T cells of a similar size, chosen at random. These plots provide a visual impression of the variation in the sizes of the clones over time and show that the sizes of clones of uninfected cells are less variable than infected cells of a similar size over periods from 1–4 years. However, the scatter plots: i) do not provide a quantitative analysis of the differences in the datasets, ii) do not include the data for most of the largest clones of infected cells, and iii) do not take full advantage of the large datasets we have for the uninfected clones (discussed below).

### Clones of infected T cells are more stable than are clones of uninfected T cells

To better address the overall stability of clones of uninfected and infected cells, we measured the differences in the sizes of each of the largest 100 clones of infected cells at two different time points and combined these differences to get an overall estimate of the stability of the clones.

**Fig 3:**
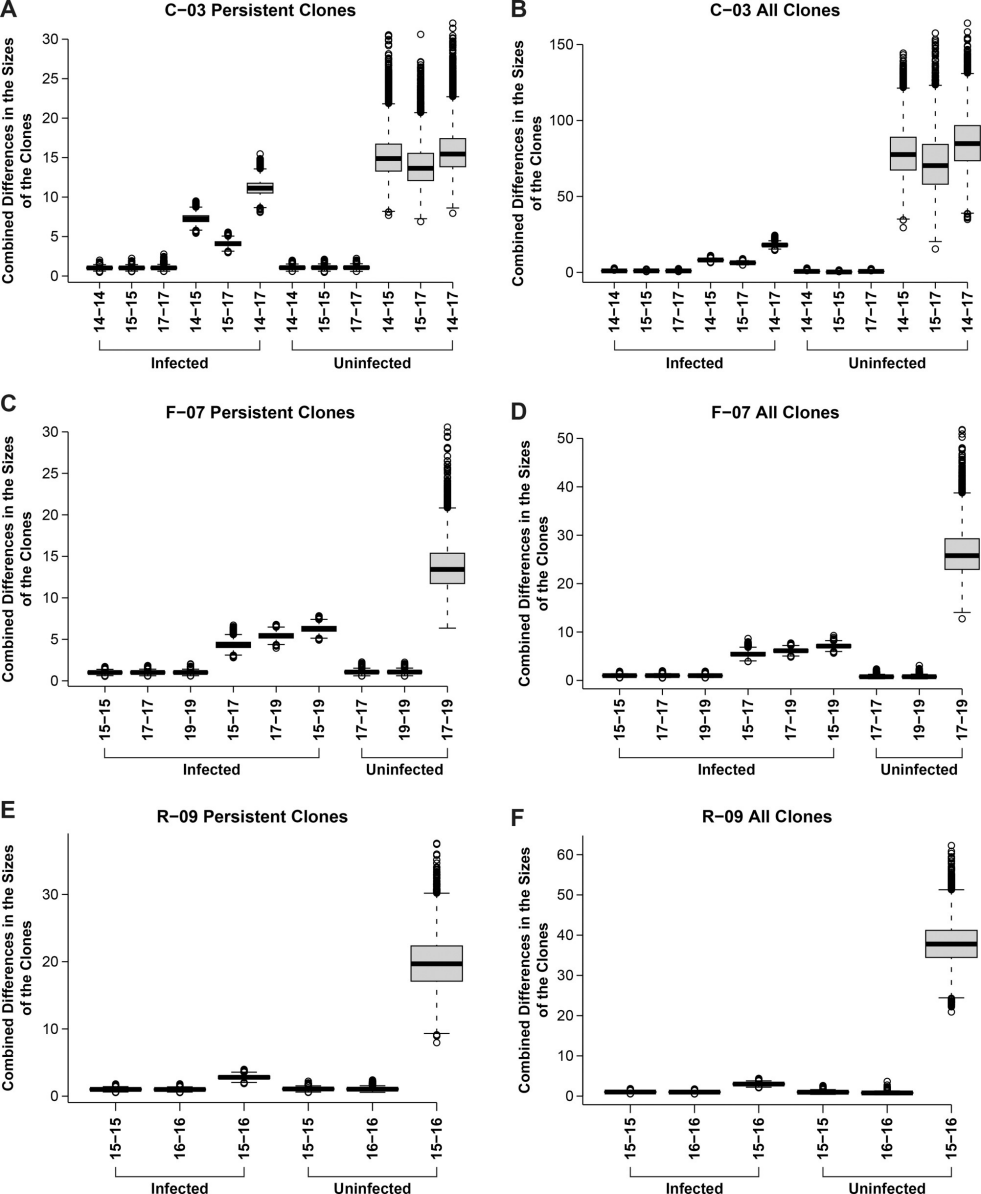
Overall stabilities of clones of infected and uninfected T cells. For each pair of time points for each donor, we determined the differences in the sizes of the each of clones in the dataset. For the comparisons of the differences in the sizes of the clones of infected cells, the 100 largest clones were used and the sizes of the clones were determined as described in the S1 Text. For the uninfected cells, a dataset of clones of a similar size was compiled, and the stability calculations were done using randomly sampled groups of 100 uninfected clones. The uncertainties in the measured sizes of the clones in each of the datasets was determined by doing a within dataset comparisons which are shown in the figure as comparisons for the same time points. The uncertainties within each of the datasets and between the datasets are shown both as a box plot (which shows the standard deviation) and as the 95% confidence limits (marked by horizontal lines separated by a dashed vertical line). The mean is the bar in the middle of the box plots. Any data from the 10,000 runs that falls outside the 95% confidence limits are shown as open circles. The method of calculating the uncertainties is described in the S1 Text. The size differences were combined to obtain a measure of the overall differences in the sizes of the group of clones for the time points being compared. The overall measure of the combined differences in the sizes of the clones, given on the Y axis, provide a good measure of the overall differences in the sizes of the clones, but the numbers on the Y axis do represent a simple metric.

We compared the overall stabilities of the 100 largest clones of infected cells from each donor to the stabilities of randomly selected groups of clones of uninfected cells from the same donor, and from the same time point. Because the stabilities of the uninfected clones depended on their size, the comparisons were done with clones of infected and uninfected cells that were similar in size (Fig 3). To determine the uncertainty in each set of clone size data, we randomly selected subsets of the data for each time point and compared the differences in the sizes of clones using the subsets. The subset comparisons were run 10,000 times and the results provide a measure of the uncertainties in the measured stabilities of the clones in each dataset and the overall differences between the datasets (Fig 3). Running the analysis 10,000 times allowed us to take advantage of the large amounts of data we have on the stabilities of the clones of uninfected cells and avoids any accidental bias that might arise if a particular subset of the clones of uninfected cells was used for the stability analysis.

In some cases, we noticed that a clone that was relatively large at one time was not detected at one of the other time points. A larger fraction of clones either appeared or disappeared in the uninfected cell datasets than in the infected cell datasets (possible explanations for this observation are considered in the Discussion). If the comparison of overall stabilities included the clones of infected and uninfected cells that either appeared or dissapeared, the infected T cell clones are much more stable than are the average uninfected CD4+ T cell clone of a similar size (Fig 3). To avoid any bias that including the clones that appeared and disappeared might have introduced, we also did the stability comparisons by choosing only clones that were detected at all of the time points used in the comparisons. The comparisions of these persistent clones also showed that the clones of infected cells are much more stable than the average clone of uninfected cells of a comparable size (Fig 3). Because in the unweighted analysis shown in Fig 3, the largest clones make a greater contribution to the analysis, we also performed the analysis weighting the data to reduce the contribution of the very largest clones. Although this affected the magnitude of the differences, it did not change the result: Infected clones are more stable than the uninfected clones.

### Stability of clones in the individual donors

For donor R-09 (Fig 3), there are data for two time points, taken approximately a year apart (2015 and 2016). A comparison of the combined differences in the sizes of the largest infected clones for R-09 at these two time points shows that, in a one year’s time, there was a small but detectable difference in the sizes of all the infected clones, relative to the uncertainties in the sizes of the clones at either of the time points being compared. For the other two donors, C-03 and F-07, there are larger differences in the overall (combined) stabilities of the clones of infected cells over longer time intervals (3 or 4 years, respectively). For C-03, the differences in the sizes of the largest infected clones are slightly greater for the shorter interval (2014–2015) than for longer intermediate interval (2015–2017). The differences in the sizes of the largest infected clones are greater over the full three-year period (2014–2017) than for the two intermediate intervals. Although the variability in the sizes of the uninfected clones was much greater for the uninfected cells in C-03, the pattern is the same. There was more variation between 2014–2015 than between 2015–2017 and, as expected, the greatest variation was between 2014–2017.

For F-07, the overall differences in the sizes of the infected clones appears to be less than for C-03 (note that, in Fig 3, the scales are different in panels B and D), despite the fact that the data for F-07 covers a total of 4 years, as opposed to 3 years for C-03. Donor F-07 is older (59 vs. 43) and had been on ART for a longer period of time (19 vs. 9 years) (see Discussion). The change in the sizes of the largest clones of infected cells in donor F-07 for the period 2017–2019 is smaller than for 2015–2017. As expected, the magnitude of the overall change from 2015–2019 is greater than are the changes for 2015–2017 and for 2017–2019. We only have data for the changes in the sizes of the uninfected clones for F-07 for the period 2017–2019. If we compare the data for all the clones, the sizes of uninfected clones are much more stable in F-07 than in C-03 or R-09. If we compare the data for the clones of uninfected cells that are present at all time points in the analysis (panels A, C and E of Fig 3), it appears that the stabilities of the uninfected clones are similar in F-07 and C-03, and that the clones in R-09 are the least stable. However, no matter how the comparisons are done, the infected clones are much more stable than the uninfected clones (see Discussion).

### Stability of large clones that carry intact infectious proviruses

Understanding the stability of the HIV reservoir requires more data on the stability of the clones of cells that carry intact, infectious proviruses. It has been previously suggested that clones of HIV infected cells that carry defective and intact proviruses can grow larger or smaller over time [26]. We recently reported the integration sites for three intact, infectious proviruses in three expanded CD4+ T cell clones, termed “repliclones”; one clone from each from the three donors R-09, C-03, and F-07 [5] (Table 4). Although, in an absolute sense, all of the clones that carried an infectious provirus were large, the sizes of the clones varied considerably, both among the donors and over time. The smallest of the clones, in donor R-09, was estimated to be ca. 1.6 x 10^4^ cells in 2015; the largest, in C-03, was estimated to be about 10^7^ cells in 2017. One of the clones that carried an intact, infectious provirus (in donor C-03) was the largest infected clone in the blood of the donor at the latest time point. The other two clones that carried intact proviruses were not in the 100 largest infected clones in the samples from their respective donors. An important consideration is whether there is a pattern in the behavior of the clones that carry intact, infectious proviruses that would distinguish them from clones carrying defective proviruses. We have far too few clones with intact proviruses to be able to answer this question definitely; however, we can ask whether the clones for which we have data behave in a similar way over time (all stable, all growing larger, all growing smaller).

**Table 4:** Stability of Clones that Carry Intact Infectious Proviruses.

Donor	Date of Blood Draw	Gene (Chromosome location in which provirus is integrated)	Total Cells Assayed for IS-qPCR	Clone Frequency (IS-qPCR)	Clone Frequency (ISA)
C-03	9/30/2014	ZNF268 (Chr. 12: 133,777,660)	1,468,800	0.48%	0.67%
	11/30/2015		1,756,800	2.53%	0.93%
	10/5/2017		2,378,700	6.54%	2.06%
R-09	8/6/2015	ABCA11P (Chr. 4: 425,776)	1,897,200	2.49%	0.07%
	9/14/2016		9,185,400	0.03%	0.01%
F-07	6/3/2015	ZNF721 (Chr. 4: 443,311)	818,100	<0.01%	<0.04%
	4/6/2017		5,448,000	0.18%	0.08%
	4/23/2019		2,656,800	0.13%	0.04%

Two independent methods, one based on integration site analysis and the other based on integration site-specific qPCR [27], were used to measure the sizes of clones carrying intact infectious proviruses at different time points. The data obtained by the two methods show good overall agreement; although when the data were compared, the qPCR method consistently showed that the clones with infectious proviruses represented a larger fraction of the infected cells than the integration site method (Table 4). The clone in R-09 that carries an intact infectious provirus decreased in size during the time we sampled this donor (see Table 4). In contrast, the clone that carried an infectious provirus in C-03 increased in size by about 3.5-fold over a three-year period, becoming, at the last time point, 2–6% of the infected cells in that donor, depending on which assay was used. The infectious clone in the third donor, F-07, is smaller than the clone that carried an infectious provirus in C-03. Both assay methods show that the clone grew larger between the first and the intermediate time point, and then decreased in size between the intermediate and the last time point. These data show, in at least some individuals on ART, that clones of infected cells that carry intact, infectious proviruses can wax or wane over time even after many years of successful long-term ART.

## Discussion

We used deep integration site and TCR sequence analysis to compare the sizes and stabilities of clones of uninfected and infected CD4+ T cells in donors who had been on ART for periods ranging from 9–19 years. The results show that i) the largest clones of uninfected CD4+ T cells were much larger than the largest clones of infected T cells; ii) the largest clones of infected T cells were more stable than were similar-sized clones of uninfected T cells, and iii) clones that carry infectious proviruses could expand or contract even after more than 9 years of ART.

Because the numbers of integration sites we recovered from the individual donors were large, the fraction of the integration sites that was shown to be in clones of expanded cells was high. Specifically, we show in this report that in samples from three individuals on long-term ART, approximately half the infected cells are in clones (43–58%). Having obtained almost 20,000 integration sites from each of three individual donors suggests that we can expect to detect clones of infected cells that are larger than about 10,000 cells, assuming that the cells in the infected clones are well-equilibrated between blood and the tissues. Clones of 10,000 cells are approximatley an order of magnitude smaller than the smallest clones that have been detected previously [8].

Having large datasets allowed us to accurately measure the sizes of the larger clones of infected cells, which made it possible to determine how the sizes clones vary over time. However, the requirement for having large datasets also determined the analysis we were able to do. We would have liked to have been able to fractionate the T cells for several reasons, including measuring the effects of T cell activation on clone size by sorting for resting cells and activated T cells. Unfortunately, we found, if the cell fractionations are done carefully, a large portion of the cells are lost. That loss of cells prevented us from obtaining the large numbers of (infected) cells that are needed for in-depth integration site analysis. Similarly, we would have liked to have measured the stability of clones of infected cells over periods of time longer than four years. Only leucopheresis samples have enough (infected) cells in them for us to obtain the large numbers of integration sites required to determine the stabilities of the clones. The requirement for leucopheresis samples constrained which donors, the samples, and the time intervals we could use. Fortunately, the differences in the relative stabilities of the infected T cell clones and uninfected T cell clones is so great that the dfferences are obvious in the samples we analyzed.

The size distribution of the infected clones shows a gradual and continuous decrease in clone size down to the smallest infected clones that were detected. The two very largest clones of uninfected cells were found in donor R-09. When the sizes of the largest 1000 clones were compared, both the clones of uninfected and infected cells were consistently larger in F-07, who had been on ART for the longest time, 19 years. Although, in each of the donors, the sizes of the clones (both infected and uninfected) fall off in a similar way, there are some differences in the distribution curves for the donors, and, for example, it appears that the sizes of the largest uninfected clones fall off more rapidly in donor R-09. However, because we have in-depth data for the sizes of the clones for only three donors, we cannot be sure if those apparent differences reflect anything more than donor-to-donor variation.

There are at least two reasons why the largest uninfected clones are considerably larger than the largest infected clones. First, only a small fraction of the CD4+ T cells are infected. CD4+ T cells can be infected either before or after they have expanded to form a clone [11]. If T cells are infected before they begin to expand to a significant extent, the cells that are infected are unlikely, based on simple probability, to be one of the rare T cells that is destined to become one of the largest T cell clones. Alternatively, if the initial infection happens in a T cell that is part of a clone that has already begun to expand, only a small fraction of the cells in the clone will be infected. Either way, the sizes of the largest clones of infected cells will be smaller in size than the largest uninfected clones.

It is important to state that sampling limitations mean that there is no good way to determine if the large numbers of integration sites that were obtained only once were derived from clones that are too small for us to detect, or if they were from infected cells that were present only once in vivo. Thus, it is inappropriate to claim that there are infected cells that are “singles” [28] nor is it possible to claim that essentially all the HIV infected cells in a person living with HIV are in clones [29], either using the data that are now available, or data that can reasonably be expected to be obtained [30].

The sizes of the clones of infected T cells from the three donors were, generally speaking, relatively stable over time. In samples taken a year apart from donor R-09, the changes in the sizes of the infected clones were smaller than the changes in the sizes of the clones of infected cells in samples from donors C-03 and F-07, for which the comparisons involved longer periods (3 and 4 years, respectively). One of our goals was to compare the relative stabilities of clones of infected and uninfected CD4+ T cells using samples obtained from the same donors. An unexpected complication we encountered in the analysis of the stabilities of the uninfected T cells over time (measured using TCR data) was that the largest clones of uninfected cells were more stable than were smaller clones. To resolve this problem, we compared the stabilities of clones of infected and uninfected CD4+ T cells that were similar in size. This analysis revealed that the clones of infected T cells were considerably more stable than the clones of uninfected T cells. When we initially analysed the data we noticed, over the time periods we analyzed (1–4 years), that the clones of uninfected cells were much more likely to either appear of disappear than were the cones of infected cells. To avoid having the clones of uninfected cells that either appeared or disappeared affect the analysis, we redid the analysis using only clones of uninfected cells that were present at the times being compared. This analysis also showed that the sizes of the clones of infected cells was still much more stable over periods of 1–4 years than were the clones of unifected cells of a similar size. It is important to point out that the difference in the stabilites of the infected and uninfected cells is very large. Even if there is some small uncertainty in the absolute sizes of the clones of infected and uninfected cells, it will not affect the conclusion that, in those on long term ART, clones of infected CD4+ cells are much are more stable than are clones of uninfected CD4+ T cells of a similar size.

Although we initially thought that the clones of uninfected cells might be more stable than clones of infected cells, we now think, in retrospect, that the greater stability of clones of infected T cells compared to clones uninfected T cells makes sense. The individuals who donated the samples we analyzed had all been on therapy for at least 9 years. Few if any infected cells would have been generated while the donors were on ART. Thus, all the clones of infected T CD4+ T cells we analyzed were derived from cells that had been infected for at least 9 years. Most of the clones of infected CD4+ T cells that were unstable would have become too small for us to detect, or would have been lost, during that 9-year interval. That means that most of the unstable clones would not be present when the first samples were taken for integration site analysis. Put another way, all the clones of infected cells that are present in people on long-term ART have been selected for long-term stability; however, there was no similar selection for the long-term stability of the corresponding clones of uninfected T cell which means the data for the infected cell clones all come from “old” CD4+ T cell clones. In contrast, the uninfected clones were a mixture of young and old CD4+ T cell clones.

In addition, there are substantial differences in the immune environment pre-ART and on ART that could potentially have affected the behavior of the clones. Pre-ART there were very high levels of HIV antigens present, and, depending on the donor, there could also be high levels of other viral antigens (CMV, KSHV, etc.). In most people living with HIV, ART allows the immune system to partially recover and, with that recovery, the levels of HIV and other viral antigens declines. It is unclear whether, or to what extent, the differences in the immune state and the antigenic environment of the host pre-ART and on ART could influence the relative stabilities of the clones of uninfected T cells that arise in the pre-ART and on ART environment. It is also possible that there are differences in the immune state of the host which could be a contributing factor. However, it is likely, if we had a way to pick out uninfected clones that were the same age as the infected clones, that their stabilities would be much more similar.

Because it is the clones that carry infectious proviruses that contribute to the reservoir, the behavior and stability of these clones is particularly important. Thus far, we only have data on the stability of three large clones of cells that carry infectious proviruses. Three clones are not enough to reach any broad conclusion about how the clones that carry infectious proviruses behave and more data are needed from additional clones that carry intact infectious proviruses. One of the T cell clones that carried an infectious provirus (in donor R-09) appeared to decrease in size over time. However, the large clone that carried an infectious provirus in donor C-03 increased in size, becoming, at the last time point, 2–6% of the infected cells in the blood, despite donor C-03 having been on therapy for more than 9 years. The data we have from donor F-07 suggest that, over about a four-year period, the clone that carried an infectious provirus first increased in size, and then decreased, despite the fact that this donor had been on therapy for 19 years. Data available for the three clones that carry infectious proviruses show that, although all three of the clones must all have persisted for at least 9 years, “old” clones that carry intact infectious proviruses can still change in size. Importantly, our results show that clones of CD4+ T cell that carry infectious proviruses can still increase in size, perhaps in response to antigenic or homeostatic stimulation [11, 12, 30], many years after the cell that gave rise to the clone was first infected. This suggests, even if the clones of infected cells that make up the reservoir are reduced in size by some therapeutic intervention, that at least some of the clones that carry infectious proviruses, and comprise part of the reservoir, could increase in size after an intervention, even if viral replication is completely blocked. Consequently, it is likely that specific recognition and elimination of clones of infected cells that carry intact proviruses will be required to eliminate the HIV reservoir and, if the procedures are to be successful, they may need to be applied over long time periods.

## Materials and methods

### Ethics statement

The University of Pittsburgh Institutional Review Board approved the study. All participants provided written informed consent before inclusion in the study. The clinical research staff completed enrollment procedures and all participants provided written informed consent before inclusion in the study.

### Participants, study approval, and sample collection

Samples for the current study were from a University of Pittsburgh study characterizing non-suppressible viremia under the IRB number PRO10070203 [5]. Study participants were referred from the University of Pittsburgh Medical Center HIV-AIDS Program or from the Allegheny Health Network Positive Health Clinic and were enrolled into the study at the University of Pittsburgh Clinical Trials Unit between April of 2014 and June of 2015. The University of Pittsburgh Institutional Review Board approved the study, the clinical research staff completed enrollment procedures, and all participants provided written informed consent before inclusion in the original study.

Longitudinal samples were collected at 2 or more time points as large-volume phlebotomy (100–180 mL) or leukapheresis between April of 2014 and April of 2019. Peripheral blood mononuclear cells (PBMC) were isolated by Ficoll-Paque density gradient centrifugation and stored as previously reported [20]. Total CD4+ T cells were bead purified from PBMC by negative selection using the EasySep Human CD4+ T-cell Enrichment kit (Stem Cell Technologies, Canada) as previously reported [20, 31]. Data from these samples for the current study were collected between May of 2014 and July of 2019.

### Extraction of genomic DNA

Genomic DNA was extracted from 1–2 × 10^6^ PBMC or total CD4+ T-cells per extraction as reported [5]. Cryopreserved cells were thawed at 37 C in a bead bath. An equal volume of warm RPMI 1640 medium (Lonza, Switzerland) was added dropwise to the cell suspension, and cells pelleted at 500 × g for 10 minutes at 40 C. Supernatant was removed from pelleted PBMC or total CD4+ T-cells, cells were resuspended in 600uL of lysis buffer [Final Concentration: 3.6M Guanidine Thiocyanate (Sigma-Aldrich, USA), 0.13M Dithiothreitol (Sigma-Aldrich), 0.67mg/mL Glycogen (Roche Applied Science, USA), 34mM N-Lauroylsarcosine (Sigma-Aldrich, USA), 34mM Sodium Citrate (Sigma-Aldrich, USA)] and incubated for 10 minutes at room temperature, followed by addition of 600uL of 100% isopropanol (Sigma-Aldrich, USA). The cell lysate was mixed by inversion and genomic DNA was pelleted by centrifugation at 21,000 × g for 15 minutes at room temperature. Liquid was removed, the genomic DNA pellet was washed with 1mL of cold 70% Ethanol (VWR, USA), and the dislodged pellet was centrifuged at 21,000 × g for 10 minutes at room temperature. After centrifugation, residual 70% ethanol was consolidated in the bottom of the tube by centrifugation at 21,000 × g for 5 minutes at room temperature and removed using a pipette. The genomic DNA was air dried for 5 minutes and resuspended in 40uL of 5mM TRIS-HCl, pH 8.0 (Thermo Fisher Scientific, USA). Extracted genomic DNA was stored at -20 C until use in the integration site analyses.

### Integration site analysis

HIV integration site analysis was carried out as previously described [1, 19]. Briefly, genomic DNAs prepared from either PBMCs or CD4 cells was sheared, end-repaired and a single dA was added to the 3’ ends of the genomic DNA fragments. In the linker ligation step, the single dA was annealed with a T-linker that has a T overhang, is composed of a long and a short oligonucleotide, and carries a 10-bp UMI. The UMI is a random sequence that is used to help determine if two or more amplified segments do (or do not) originate from different original pieces of host DNA. NEBNext Ultra II DNA Library Prep Kit for Illumina (New England Biolabs, Cat No. E7645S) was used for the fragmentation and ligation steps. Ligated DNA was further purified by Agencourt AMPure XP system (Beckman Coulter, Cat No. A63881). The DNA fragments were subjected to two rounds of PCR amplifications. Primers were designed to amplify the DNA fragments carrying the HIV LTRs and linkers. The initial round of PCR, when combined with a nested round, generated DNA products with good quantity and specificity. After each PCR amplification step, PCR products were subjected to purification steps using Agencourt AMPure XP system and QC steps via qRT-PCR, Qubit fluorometric quantification (Thermo Fisher Scientific) or TapeStation System (Agilent Technologies, Inc.). The PCR products were sequenced using the Illumina MiSeq system.

Fastq files were generated from the sequencing data. There are a series of steps used in our data pipeline [19]. In general, data were partitioned into different groups based on indexes designed in the primers. Reads were trimmed to remove indexes, primer sequences, and linker sequences. Short reads and reads with low quality were removed. Reads with high quality were then mapped to Homo sapiens genome assembly hg19 from the Genome Reference Consortium by BLAT (standalone BLAST-like alignment tool, v. 35x1 fast sequence search command line tool). After the alignment, several filter steps were used to remove reads mapping to multiple places in the genome and reads with low mapping quality. Steps were included in the pipeline that used the UMIs to distinguish reads with real breakpoints and ambiguous reads resulted from sequencing errors and any possible contamination. A finalized report was generated in standard format listing all of the HIV integration sites recovered and identifying genes that had integrated proviruses.

In order to compare the integration sites that were in clones in samples from different patients, different cell types and different extraction dates, data were partitioned into different sets of groups and subjected to clone calling. Clone calling was the same as described in our previous study [8]. Briefly, for samples derived from patients on long-term ART, we consider that integration sites detected twice with different breakpoints have undergone clonal expansion. We do use different criteria for calling clones in untreated patients; that protocol does not apply to the data obtained in this study.

### Integration Site-Specific qPCR

Integration site-specific qPCR was performed as described [27]. In brief, for each of the targeted proviruses, a region spanning the host-virus junction was selected based on the chromosome location of the integration site and orientation of the provirus of interest. We used sequence data previously obtained [5] to design primers and probes specific to the provirus of interest. Forward primers were designed immediately upstream (26–113 bp) of the 5’ host-virus junction, and the reverse primer immediately downstream (49–94 bp) in U3. The probe sequences spanned the host-virus junction and were labeled with a 5’ FAM fluorophore, a 3’ dark quencher, and an additional internal ZEN quencher (IDT, USA). Forward and reverse primers and probe sequences, 5’-3’ respectively, are listed as follows: R-09 TGCCTTTGTGCTTTAAGAAACTC, GAAGTAGCCTTGTGTGTGGTAG, AGATGGTATGTACCCTGGAAGGGCT; C-03 GAGTCTGGAATTGAATACATG, ACAAATCAAGGATGTCTTGTCT, CCAAACTAGCCCTTCCAAGTATACAGTGAT; F-07 GAATGCTTTACCACACAGTAC, GTGTAGTTCTGCCAATCAG, AGCTGGAAGGGCTAGTTTACTCCCAG. Cycling parameters for real-time PCR were 95°C for 5 minutes, followed by 95°C for 15 seconds and 60°C for 1 minutes, for 50 cycles of amplification.

To determine the number of cell equivalents in the PCR reactions, nucleic extract was quantified in duplicate or triplicate by qPCR for the CCR5 gene, according to a previously published protocol [20, 32]. To assess the total number of LTRs in a given sample, nucleic extract was quantified in duplicate or triplicate by qPCR for the R/U3 region of LTR. To count proviruses, the number of LTR copies was divided by two. Forward and reverse primers with modified degenerate bases [33] (LGC Biosearch Technololgies) were designed to account for observed single nucleotide polymorphisms at these locations (analysis from Los Alamos National Laboratory HIV Sequence Database): FWD G/idK/GAACCCACTGCTTAAG and REV GTCTGAGGGATCTCTAG/idP/TAC. These primers were used in tandem with a minor groove binder (MGB) modified probe (ThermoFisher Scientific): 6FAM-TCAATAAAGCTTGCCTTGAGTG-MGBNFQ. Reaction and cycling parameters were the same as those described above. Copies of the LTR standard were quantified using an in-run standard corresponding to position 87 through 8680 (HXB2 numbering) constructed by amplification of proviral DNA from the JLat 6.3 cell line (AIDS Reagent Database). Clone frequencies were reported as the copies of a particular provirus to the total LTR copies.

### Cell Sorting for TCR analysis

Thawed cells were stained with diluted LIVE/DEAD discrimination dye and subsequently stained for 15 minutes at room temperature with titrated amounts of anti-human CD3 Cy7-H7, anti-human CD4 PE-Cy5.5, and anti-human CD45RO ECD. Cells were washed once with 1X DPBS and resuspended in complete RPMI for sorting. Stained cells were sorted for live memory CD4 T cells using a BD S6 sorter the gating scheme: lymphocytes, singlets, live CD3+ cells, CD4+CD45RO+ cells.

Antibodies & Dyes: Anti-human CD3 APC-H7, clone SK7 –BD Biosciences–Cat#: 641397–1:30 dilution; Anti-human CD4 PE-Cy5.5, clone S3.5 –ThermoFisher Scientific–Cat#: MHCD0418–1:200 dilution; Anti-human CD45RO ECD, clone UCHL1 –Beckman Coulter–Cat# IM2712U – 1:30 dilution; LIVE/DEAD Fixable Violet Dead Cell Stain Kit, for 405nm excitation–ThermoFisher Scientific–Cat#: L34955–1:40 Dilution.

### TCR analysis

T cell receptor genes were sequenced using a method similar to that described in Gros et al. [18] In brief, extracted RNA was denatured and cDNA was synthesized using an oligo (dT) primer and a template-switching oligo during an incubation at 42 C for 90 minutes then 70 C for 10 minutes. Following purification with AMPure XP beads (Beckman Coulter), cDNA was amplified using the 5PIIA primer (5’- AAGCAGTGGTATCAACGCAGAGT-3’) and constant region primers TCRb (5’-TGCTTCTGATGGCTCAAACACAGCGACCT-3’) or TCRa (5’-TCTCAGCTGGTACACGGCAGGGTCAGGGT-3’) with the following cycling conditions: (95 C 5 min, 5 cycles of 98 C 15 sec, 72 C 1 min, 5 cycles of 98 C 15 sec, 70 C 10 sec, 72 C 1 min, 10–15 cycles of 98 C 15 sec, 68 C 10 sec, 72 C 1 min). The amplicons were purified using AMPure XP beads, amplified further to incorporate Illumina sequences, and subsequently sequenced by paired end MiSeq 2x151 base pair reads. Paired end TCRb reads were annotated using MiXCR (v3.0.6) [34] with default settings. Clonotypes with counts less than 2 were discarded as likely sequencing or PCR errors.

## Supporting information

S1 Table Integration Sites. (XLSX)

S1 Text Data Analysis. (DOCX)

S1 Fig Stabilities of individual clones of infected and uninfected cells from the three donors. For each donor, the data for the largest 100 clones of infected T cells are shown for either two or three time points. Stability data are also shown for the same number of clones of uninfected T cells of a similar size, chosen at random from the much larger TCR dataset. Because a significant fraction of the clones of uninfected cells were not detected at all the time points, we also analyzed the stabilities of clones of uninfected T cells for each donor using only data for clones of uninfected cells that were present at all the time points in the analysis. For F-07, there are data for the infected T cells clones from three time points. For the uninfected cells, there are data for only two of those time points.
(TIF)
