# Supplementary material for: HIV infected CD4+ T cell clones are more stable than uninfected clones during long-term antiretroviral therapy
Source: PLoS Pathog. 2022 Aug 31;18(8):e1010726. doi: 10.1371/journal.ppat.1010726 (PMC9432747; doi:10.1371/journal.ppat.1010726)
Supplement: S1 Text — (DOCX) [file ppat.1010726.s002.docx]

Supplementary Materials

**S1 Text**

**Calculating the Differences in Clone Sizes Over Time (Computational Analysis)**

The data can be used to determine the sizes of each of the clones of infected and uninfected cells. Finding the integration for a particular provirus many times at a given time point strongly suggests that the clone whose cells carry this provirus is large, while a clone for which many fewer integration sites were detected is expected to be much smaller. For each donor, the number of observed insertion sites was scaled so that their sizes sum to the total number of infected T cells present in the donor at that time point (see also the main text). The number of infected T cells was calculated based the fraction of the CD4+ T cells that were infected and the numbers of CD4+ T cell present in each of the three donors. Averaging the sizes of each of the clones across all of the time points produces a time-averaged size, and, to ensure that we have enough integration site data to accurately determine the sizes of the clones only the 100 largest time-averaged clones were considered (see below). Using the average sizes of the clones has an important advantage: If we choose the largest clones based on their size at the first timepoint, we bias the data toward those clones that are large early and became smaller. Conversely, if we choose the largest clones based on the last timepoint, we bias the data towards the clones that start small and become larger. Choosing the largest clones by their average size avoids both of these biases.

A similar approach was used for the clones of uninfected CD4+ T cells. For each donor and each time point, the uninfected cell clones were scaled to sum to the total CD4+ T cells present in that donor, and the time-averaged size for each clone was determined. The sizes of the 100 largest time-averaged clones at each time point for the infected and uninfected cells is shown in S1 Fig., and the clones are ranked in decreasing order according to their sizes. None of the curves decrease monotonically for any given time point; the sizes only decrease monotonically for the time-averaged sizes of the clones, not for the sizes of the clones at any given time.

To calculate the differences in the sizes of the clone of infected cells for each donor within or across time points, the following procedure was used. For each donor, and for the two time points being compared, 2000 infected cells were probabilistically selected from among the 100 largest time-averaged clones. This was done using a roulette wheel approach, which is a proportional selection method. This procedure is a common selection method in population-based global optimization procedures, like a genetic algorithm [1]. A model roulette wheel was constructed for each time point, where the size of the wedge in each of the 100 sections was equal to the fractional size of each clone across the set of 100 clones. The integration sites from 2000 infected cells were selected as the sample size because the number of infected cells whose integration sites were determined for C-03 in 2014 and F-07 in 2015 were 2077 and 2516, respectively, and we did not want the sampling sizes to exceed the number of observed infected cells for any donor or time point. To determine the variability due to sampling, a single roulette wheel representing a donor and time point was used to select two sets of 2000 infected cells. If the comparison is between two time points, two separate roulette wheels representing each time point was used to select 2000 infected cells from each time point.

For each set, the sizes of each of the 100 clones is determined; the difference in sizes of each clone at the two time points can be displayed as a scatter plot. A regression line was constructed and the perpendicular distance between the point in the scatter plot and the regression line represents the difference in the size of each clone between the two sets. Because larger clones should have a larger distance to the regression line, the difference metric used here is the perpendicular distance to the regression line squared divided by the average size of this clone in the two sets. The average of this relative squared distance across all 100 clones then represents the difference in the clonal size distribution across the two sets of 2000 integration sites. This procedure is repeated 10,000 times to capture the variability of selecting 2000 integration sites across this set of 100 largest overall clones.

For the clones of uninfected T cells, the procedure is slightly different in that all the clones of uninfected cells with a time-averaged size that is comparable to the range of time-averaged sizes for the 100 largest infected clones were considered. For C-03, for example, the 100 largest clones of infected cells have a time-average size that ranges from 10,036,444 to 619,487. A total of 45,429 clones of uninfected T cells span this range of time-averaged sizes. Using this set of 45,429 uninfected clones, a master roulette wheel was constructed using the fraction of the total that the sizes of these clones represent. In each trial, 100 clones are selected using this master roulette wheel and those 100 clones were then used to construct a secondary roulette wheel for each time point. If the uninfected clones from C-03 in 2014 were compared to themselves, only a single secondary roulette wheel was constructed. To compare the sizes of the uninfected clones at two different time points, two secondary roulette wheels were constructed from the sets of 100 selected clones of uninfected cells. 2000 TCR sequences were chosen using the appropriate secondary roulette wheel. Those data were used to determine the sizes of the uninfected clones for each time point and the average relative distance squared to the regression line was determined. For the next trial a different set of 100 TCR clones was selected from the master roulette wheel, and this process was repeated 10,000 times.

The changes in the sizes of the infected and uninfected clones that were detected at all the time points for C03, F-07, and R-09 were also analyzed. These will be referred to as persistent clones. In addition, because the sizes of the 100 largest overall integration site clones drops off very quickly, the scatter plots are dominated by points relatively close the origin. To increase the fit of the regression line to the larger clones, a weighted regression was also used where the weight was equal to the mean of the clone sizes for the time points being examined. The results using the unweighted and weighted regression lines for the 100 largest clones of infected cells and for the comparably sized uninfected clones both showed that the clones of infected cells were much more stable that were similar sized clones of uninfected cells.

**Effect of Reduced Sampling on Measured Clonal Structure**

The table below shows the overall number of integration sites for the three donors

| **Donor** | **Number of Time Points** | **Total IS** | **Unique IS** | **#Clones** | **Number of IS in Clones** | **Percent of IS in Clones** |
| --- | --- | --- | --- | --- | --- | --- |
| C-03 | 3 | 19712 | 12033 | 1653 | 8856 | 44.9 |
| F-07 | 3 | 20185 | 12225 | 1301 | 8747 | 43.3 |
| R-09 | 2 | 19230 | 9596 | 1768 | 11197 | 58.2 |

The data for the integration sites clones of infected cells was combined across all time points for each donor, producing three datasets with approximately 20,000 integration sites from each donor. The numbers of times each integration site was recovered was divided by the total number of integration sites to produce the probability that the integration site for a particular clone would be recovered if a smaller number of integration sites were analyzed. For each individual, 10,000 sampling runs were performed in which either 1,000 or 500 integration sites were selected based on the probability distribution for each integration site.

In this analysis, the structure of the 10 largest clones of infected cells in the original dataset was analyzed, although for the analysis in the main text the 100 largest clones of infected cells were analyzed to determine the overall difference in the sizes of the clones at different times. For the 95% confidence interval (CI) for the size of the clones and ratio of the size of the N^th^ clone to the first (N = 2, 3, …, 10), the 10,000 values are sorted by value and the smallest and largest 250 values are removed. For the number of times a clone was not observed and the number of times the N^th^ clone was larger than the first, all 10,000 trials are used.

**Donor C-03**

The observed size, fractional size, size ratio relative to the largest clone of infected cells (C(1)/C(N)) and expected size from 1000 and 500 draws are shown in the following table:

| **Observed Size** | **Fractional Size** | **C(1)/C(N)** | **Expected (1000)** | **Expected (500)** |
| --- | --- | --- | --- | --- |
| 279 | 0.014 | --- | 14.15 | 7.08 |
| 128 | 0.0065 | 2.18 | 6.49 | 3.25 |
| 99 | 0.0050 | 2.82 | 5.02 | 2.51 |
| 93 | 0.0047 | 3 | 4.72 | 2.36 |
| 90 | 0.0046 | 3.1 | 4.57 | 2.28 |
| 70 | 0.0036 | 3.99 | 3.55 | 1.78 |
| 62 | 0.0031 | 4.5 | 3.15 | 1.57 |
| 56 | 0.0028 | 4.98 | 2.84 | 1.42 |
| 56 | 0.0028 | 4.98 | 2.84 | 1.42 |
| 51 | 0.0026 | 5.47 | 2.59 | 1.29 |

As shown in the table above, C-03 has one clone that is 2.18 times larger than the next largest clone and 5.47 times larger than the 10^th^ largest clone. These 10 clones contain 4.99% of all observed insertion sites.

The results from the 10,000 experiments that used sample sizes of 1000 and 500 are shown below.

|  | **Sample Size = 1000** | | | |  | **Sample Size = 500** | | | |
| --- | --- | --- | --- | --- | --- | --- | --- | --- | --- |
| **Clone** | **95% CI**  **(Observed)** | **95% CI**  **(C(1)/C(N))** | **Unobserved** | **Times**  **C(1)≤C(N)** |  | **95% CI**  **(Observed)** | **95% CI**  **(C(1)/C(N))** | **Unobserved** | **Times**  **C(1)≤C(N)** |
| 1 | 7 to 22 | 1.0 to 1.0 | 0 | --- |  | 2 to 12 | --- | 8 | --- |
| 2 | 2 to 12 | 0.889 to 7.5 | 17 | 564 |  | 0 to 7 | 0.571 to Inf* | 419 | 1533 |
| 3 | 2 to 11 | 0.909 to 9 | 24 | 452 |  | 0 to 7 | 0.625 to Inf | 469 | 1306 |
| 4 | 2 to 11 | 1 to 9.5 | 36 | 369 |  | 0 to 7 | 0.667 to Inf | 604 | 1198 |
| 5 | 1 to 10 | 1.1 to 14 | 89 | 218 |  | 0 to 6 | 0.714 to Inf | 870 | 866 |
| 6 | 1 to 9 | 1.143 to 15 | 102 | 174 |  | 0 to 6 | 0.75 to Inf | 1009 | 794 |
| 7 | 1 to 9 | 1.167 to 16 | 99 | 168 |  | 0 to 6 | 0.75 to Inf | 1023 | 764 |
| 8 | 0 to 8 | 1.4 to Inf | 277 | 53 |  | 0 to 5 | 1.0 to Inf | 1687 | 453 |
| 9 | 0 to 7 | 1.6 to Inf | 452 | 37 |  | 0 to 4 | 1.0 to Inf | 2131 | 357 |
| 10 | 0 to 7 | 1.714 to Inf | 629 | 20 |  | 0 to 4 | 1.0 to Inf | 2515 | 296 |

*Inf means infinity

When the sample size is 1000, there is a 6.29% chance that the 10^th^ largest clone of infected cells will not be observed. When the sample size drops to 500, there is a 4.19% chance that the 2^nd^ largest clone is not seen and a 25.15% chance that the 10^th^ largest clone will not be observed. There is also a 2.96% chance that the 10^th^ largest clone will be scored as being at least as large as the largest clone, even though the largest clone is 5.47 times larger. The following table shows the number of times at least one of the 10 largest clones were not detected in 10,000 trials.

| **Number**  **Missing** | **1000** | **500** |
| --- | --- | --- |
| 5 | 0 | 16 |
| 4 | 0 | 127 |
| 3 | 5 | 602 |
| 2 | 100 | 2160 |
| 1 | 1510 | 4020 |
| Total | 1615 | 6925 |

For a sample size of 1000, there is a 16.15% chance that one or more of the 10 largest clones of infected cells will not be observed. When the sample size is reduced to 500, there is a 69.25% chance that at least one of the 10 largest clones will not be observed and a 6.02% chance that three of them will be missed.

**Donor F-07**

The observed size, fractional size, size ratio relative to the largest clone of infected cells (C(1)/C(N)) and expected size from 1000 and 500 draws are shown in the following table.

| **Observed Size** | **Fractional Size** | **C(1)/C(N)** | **Expected (1000)** | **Expected (500)** |
| --- | --- | --- | --- | --- |
| 420 | 0.021 | --- | 20.81 | 10.40 |
| 385 | 0.019 | 1.09 | 19.07 | 9.537 |
| 230 | 0.011 | 1.83 | 11.39 | 5.70 |
| 145 | 0.0072 | 2.90 | 7.18 | 3.591 |
| 108 | 0.0054 | 3.89 | 5.35 | 2.68 |
| 96 | 0.0048 | 4.38 | 4.76 | 2.38 |
| 96 | 0.0048 | 4.38 | 4.76 | 2.38 |
| 87 | 0.0043 | 4.828 | 4.31 | 2.16 |
| 80 | 0.0040 | 5.25 | 3.96 | 1.98 |
| 78 | 0.0039 | 5.38 | 3.86 | 1.932 |

In contrast to C-03, F-07 has two large clones of infected cells, both of which are fractionally larger than the largest C-03 clone. The 10 largest clones in F-07 contain 8.55% of all observed insertion sites.

The results from the 10,000 experiments that used sample sizes of 1000 and 500 are shown below.

|  | **Sample Size = 1000** | | | |  | **Sample Size = 500** | | | |
| --- | --- | --- | --- | --- | --- | --- | --- | --- | --- |
| **Clone** | **95% CI**  **(Observed)** | **95% CI**  **(C(1)/C(N))** | **Unobserved** | **Times**  **C(1)≤C(N)** |  | **95% CI**  **(Observed)** | **95% CI**  **(C(1)/C(N))** | **Unobserved** | **Times**  **C(1)≤C(N)** |
| 1 | 12 to 30 | --- | 0 | --- |  | 5 to 17 | ---- | 0 | --- |
| 2 | 11 to 27 | 0.57 to 2.13 | 0 | 4253 |  | 4 to 16 | 0.41 to 3.0 | 1 | 4626 |
| 3 | 5 to 18 | 0.88 to 4.33 | 0 | 585 |  | 2 to 11 | 0.63 to 7.5 | 46 | 1504 |
| 4 | 2 to 13 | 1.31 to 9 | 10 | 49 |  | 0 to 8 | 1.0 to Inf* | 273 | 457 |
| 5 | 1 to 10 | 1.67 to 17 | 51 | 12 |  | 0 to 6 | 1.2 to Inf | 727 | 181 |
| 6 | 1 to 9 | 1.88 to 22 | 101 | 5 |  | 0 to 6 | 1.29 to Inf | 938 | 136 |
| 7 | 1 to 9 | 1.88 to 22 | 99 | 4 |  | 0 to 6 | 1.33 to Inf | 904 | 122 |
| 8 | 1 to 9 | 2 to 24 | 150 | 5 |  | 0 to 5 | 1.4 to Inf | 1201 | 105 |
| 9 | 1 to 8 | 2.14 to 28 | 205 | 2 |  | 0 to 5 | 1.5 to Inf | 1405 | 80 |
| 10 | 0 to 8 | 2.14 to Inf | 251 | 5 |  | 0 to 5 | 1.5 to Inf | 1468 | 74 |

*Inf means infinity

While the ratio of clone sizes for the two largest clones of infected cells is only 1.09, this ratio varies between 0.567 and 2.133 when 1000 IS are used, and between 0.412 and 3.0 for the sets using 500 IS (95% CI). The 10 largest clones are seen at least once in many more trials using 500 IS than for C-03, but the 8^th^, 9^th^, and 10^th^ largest clones are still missing from between 12.01% and 14.68% of the simulations. The following table shows the number of times at least one of the 10 largest clones was not observed in the 10,000 trials.

| **Number Missing** | **1000** | **500** |
| --- | --- | --- |
| 5 | 0 | 1 |
| 4 | 0 | 19 |
| 3 | 0 | 232 |
| 2 | 25 | 1217 |
| 1 | 817 | 3752 |
| Total | 842 | 5221 |

If 1000 insertion sites are examined there is an 8.42% chance that one or two of the 10 largest clones will not be observed. If only 500 insertion sites are identified, there is a 52.21% chance that at least one the 10 largest clones will not be observed. These probabilities are smaller than for C-03, but still substantial, especially when only 500 insertion sites are identified.

**Donor R-09**

The observed size, fractional size, size ratio relative to the largest clone (C(1)/C(N)) and expected size from 1000 and 500 draws are shown in the following table.

| **Observed Size** | **Fractional Size** | **C(1)/C(N)** | **Expected (1000)** | **Expected (500)** |
| --- | --- | --- | --- | --- |
| 295 | 0.015 | --- | 15.34 | 7.67 |
| 205 | 0.011 | 1.44 | 10.66 | 5.33 |
| 185 | 0.0096 | 1.59 | 9.62 | 4.81 |
| 153 | 0.0080 | 1.93 | 7.96 | 3.98 |
| 150 | 0.0078 | 1.97 | 7.80 | 3.90 |
| 133 | 0.0069 | 2.22 | 6.92 | 3.46 |
| 123 | 0.0064 | 2.40 | 6.40 | 3.20 |
| 99 | 0.0051 | 2.987 | 5.15 | 2.57 |
| 94 | 0.0049 | 3.14 | 4.89 | 2.44 |
| 84 | 0.0044 | 3.51 | 4.37 | 2.18 |

The largest R-09 clone is 43.9% larger than the second largest, and overall, these 10 clones contain 7.91% of all of the observed integration sites. This is more than for C-03, but less than F-07. It should be noted that for C-03 and F-07 the largest clone is about 5.5 and 5.4 times larger than the 10^th^ largest clone, respectively, while for R-09 this ratio is only about 3.5, showing a smaller reduction in clone sizes across the 10 largest.

The results from the 10,000 experiments that used sample sizes of 1000 and 500 are shown below.

|  | **Sample Size = 1000** | | | |  | **Sample Size = 500** | | | |
| --- | --- | --- | --- | --- | --- | --- | --- | --- | --- |
| **Clone** | **95% CI**  **(Observed)** | **95% CI**  **(C(1)/C(N))** | **Unobserved** | **Times**  **C(1)≤C(N)** |  | **95% CI**  **(Observed)** | **95% CI**  **(C(1)/C(N))** | **Unobserved** | **Times**  **C(1)≤C(N)** |
| 1 | 7 to 21 | --- | 0 | --- |  | 3 to 13 | --- | 3 | --- |
| 2 | 4 to 16 | 0.59 to 3.57 | 1 | 2402 |  | 1 to 10 | 0.429 to 6 | 51 | 3037 |
| 3 | 3 to 14 | 0.69 to 4.67 | 3 | 1523 |  | 1 to 9 | 0.5 to 8 | 81 | 2592 |
| 4 | 3 to 13 | 0.75 to 5.5 | 7 | 1099 |  | 1 to 8 | 0.556 to 12 | 208 | 1805 |
| 5 | 3 to 12 | 0.78 to 6 | 10 | 898 |  | 1 to 8 | 0.571 to 11 | 177 | 1675 |
| 6 | 2 to 11 | 0.88 to 8.5 | 18 | 553 |  | 0 to 7 | 0.667 to Inf* | 312 | 1351 |
| 7 | 2 to 11 | 0.89 to 8 | 21 | 564 |  | 0 to 7 | 0.667 to Inf | 439 | 1159 |
| 8 | 1 to 10 | 1 to 11 | 56 | 309 |  | 0 to 6 | 0.8 to Inf | 784 | 722 |
| 9 | 1 to 9 | 1.13 to 15 | 92 | 194 |  | 0 to 6 | 0.8 to Inf | 861 | 640 |
| 10 | 1 to 8 | 1.29 to 19 | 184 | 103 |  | 0 to 5 | 1.0 to Inf | 1196 | 533 |

*Inf means infinity

Even though the largest clone is 43.9% larger than the second, this second clone was scored as being at least as large as the first 20.57 and 30.37% of the time when the sample size was 1000 and 500, respectively. The number of times a clone was not observed was smaller for R-09 than either C-03 or F-07, but the 10^th^ largest clone was not detected 11.96% of the time when the sample size is 500.

The following table shows the number of times at least one of the 10 largest clones was not observed in the 10,000 trials.

| **Number Missing** | **1000** | **500** |
| --- | --- | --- |
| 5 | 0 | 0 |
| 4 | 0 | 1 |
| 3 | 0 | 39 |
| 2 | 4 | 529 |
| 1 | 281 | 2933 |
| Total | 285 | 3502 |

Though there was a reduction in the probability of missing one or more of the 10 largest clones of infected cells, there was still a 35.02% chance one or more of these clones was missed when a sample size of 500 was used.

Understand that the 95% confidence intervals for each large clone may be non-overlapping sets. In other words, a run that has a clone size for the largest clone within the 95% CI may have a clone size for the second largest (or the third, etc.) that is outside of its 95% CI.

To really understand the difference between two clonal distributions, for example to determine if there is a change in clone sizes over one or more years, more than the largest 10 clones need to be examined. In the tables below changes in the largest 10 clones, the largest 20 clones, up to the largest 100 clones are examined, assuming sample sizes of 1000 and 500 insertion sites. Once again, the 95% CI for the number of insertion sites contained in the N largest clones and the 95% CI for number of unidentified clones do not necessarily represent the same set of 9500 trials, but they will give an indication of the variability that would be observed.

**Donor C-03**

**Sample Size = 1000**

|  | **Number of Integration Sites** | | | |  | **Missed** | | |
| --- | --- | --- | --- | --- | --- | --- | --- | --- |
| **Top** | **Expect** | **Min** | **Max** | **95% CI** |  | **Min** | **Max** | **95% CI** |
| 10 | 49.92 | 32 | 86 | 42 to 70 |  | 0 | 3 | 0 to 1 |
| 20 | 70.41 | 50 | 113 | 62 to 96 |  | 0 | 6 | 0 to 4 |
| 30 | 86.04 | 64 | 130 | 77 to 114 |  | 0 | 11 | 0 to 7 |
| 40 | 98.57 | 76 | 148 | 90 to 129 |  | 0 | 16 | 2 to 10 |
| 50 | 109.17 | 86 | 159 | 101 to 142 |  | 1 | 19 | 4 to 14 |
| 60 | 118.20 | 94 | 174 | 111 to 152 |  | 3 | 24 | 7 to 18 |
| 70 | 126.32 | 104 | 184 | 119 to 162 |  | 5 | 29 | 10 to 23 |
| 80 | 133.22 | 111 | 191 | 128 to 172 |  | 8 | 34 | 13 to 27 |
| 90 | 139.20 | 117 | 197 | 135 to 179 |  | 11 | 39 | 17 to 33 |
| 100 | 144.78 | 122 | 203 | 141 to 187 |  | 15 | 46 | 22 to 38 |

Even if only the 40 largest clones are considered, at least two and as many as 10 of these largest clones are likely to be missed. It is also interesting to note that the expected number of insertion sites in each set of clones lies at the low end of the 95% confidence interval.

**Donor C-03**

**Sample Size = 500**

|  | **Number of Integration Sites** | | | |  | **Missed** | | |
| --- | --- | --- | --- | --- | --- | --- | --- | --- |
| **Top** | **Expect** | **Min** | **Max** | **95% CI** |  | **Min** | **Max** | **95% CI** |
| 10 | 24.96 | 12 | 50 | 18 to 38 |  | 0 | 5 | 0 to 3 |
| 20 | 35.21 | 17 | 65 | 28 to 51 |  | 0 | 11 | 1 to 8 |
| 30 | 43.02 | 25 | 73 | 35 to 61 |  | 1 | 17 | 4 to 13 |
| 40 | 49.28 | 28 | 82 | 41 to 68 |  | 4 | 25 | 8 to 19 |
| 50 | 54.59 | 33 | 88 | 47 to 75 |  | 7 | 31 | 13 to 25 |
| 60 | 59.10 | 41 | 93 | 51 to 81 |  | 11 | 40 | 18 to 32 |
| 70 | 63.16 | 46 | 99 | 56 to 86 |  | 18 | 46 | 24 to 39 |
| 80 | 66.61 | 47 | 104 | 59 to 91 |  | 23 | 53 | 30 to 46 |
| 90 | 69.60 | 48 | 110 | 63 to 95 |  | 28 | 61 | 36 to 53 |
| 100 | 72.39 | 52 | 117 | 66 to 99 |  | 32 | 70 | 43 to 61 |

Using only 20 largest clones in a comparison could cause a problem because between 1 and 8 of the largest clones may be missed.

**Donor F-07**

**Sample Size = 1000**

|  | **Number of Integration Sites** | | | |  | **Missed** | | |
| --- | --- | --- | --- | --- | --- | --- | --- | --- |
| **Top** | **Expect** | **Min** | **Max** | **95% CI** |  | **Min** | **Max** | **95% CI** |
| 10 | 85.46 | 51 | 120 | 67 to 101 |  | 0 | 2 | 0 to 1 |
| 20 | 111.96 | 75 | 151 | 91 to 129 |  | 0 | 5 | 0 to 3 |
| 30 | 129.75 | 90 | 164 | 107 to 148 |  | 0 | 10 | 0 to 6 |
| 40 | 145.06 | 103 | 184 | 121 to 164 |  | 0 | 13 | 1 to 9 |
| 50 | 158.34 | 113 | 202 | 133 to 178 |  | 0 | 17 | 3 to 12 |
| 60 | 169.58 | 121 | 215 | 143 to 190 |  | 2 | 21 | 6 to 17 |
| 70 | 179.59 | 128 | 223 | 153 to 200 |  | 3 | 26 | 9 to 21 |
| 80 | 188.36 | 134 | 236 | 161 to 209 |  | 7 | 32 | 12 to 26 |
| 90 | 196.43 | 139 | 248 | 168 to 212 |  | 10 | 36 | 16 to 31 |
| 100 | 204.062 | 147 | 253 | 175 to 225 |  | 14 | 43 | 20 to 36 |

**Donor F-07**

**Sample Size = 500**

|  | **Number of Integration Sites** | | | |  | **Missed** | | |
| --- | --- | --- | --- | --- | --- | --- | --- | --- |
| **Top** | **Expect** | **Min** | **Max** | **95% CI** |  | **Min** | **Max** | **95% CI** |
| 10 | 42.73 | 20 | 68 | 30 to 54 |  | 0 | 5 | 0 to 3 |
| 20 | 55.98 | 32 | 84 | 42 to 69 |  | 0 | 9 | 1 to 7 |
| 30 | 64.87 | 39 | 93 | 50 to 79 |  | 0 | 16 | 4 to 12 |
| 40 | 72.53 | 42 | 102 | 56 to 87 |  | 2 | 22 | 7 to 18 |
| 50 | 79.17 | 49 | 107 | 62 to 94 |  | 4 | 29 | 12 to 24 |
| 60 | 84.79 | 52 | 116 | 67 to 100 |  | 9 | 37 | 17 to 30 |
| 70 | 89.79 | 57 | 124 | 72 to 105 |  | 15 | 44 | 22 to 37 |
| 80 | 94.18 | 61 | 128 | 76 to 110 |  | 19 | 52 | 28 to 44 |
| 90 | 98.22 | 66 | 131 | 80 to 114 |  | 27 | 59 | 35 to 51 |
| 100 | 102.03 | 69 | 135 | 83 to 118 |  | 33 | 66 | 41 to 58 |

With a sample size of 1000, at least one of the 40 largest clones will not be observed and the number that are missed could be as large as 9. If the sample size is reduced to 500, between 1 and 7 of the largest 20 clones would be missed.

**Donor R-09**

**Sample Size = 1000**

|  | **Number of Integration Sites** | | | |  | **Missed** | | |
| --- | --- | --- | --- | --- | --- | --- | --- | --- |
| **Top** | **Expect** | **Min** | **Max** | **95% CI** |  | **Min** | **Max** | **95% CI** |
| 10 | 79.095 | 49 | 108 | 62 to 95 |  | 0 | 2 | 0 to 1 |
| 20 | 113.10 | 76 | 149 | 93 to 132 |  | 0 | 4 | 0 to 2 |
| 30 | 137.75 | 98 | 173 | 115 to 157 |  | 0 | 7 | 0 to 4 |
| 40 | 156.58 | 118 | 197 | 133 to 177 |  | 0 | 10 | 0 to 6 |
| 50 | 172.33 | 131 | 224 | 147 to 193 |  | 0 | 14 | 1 to 9 |
| 60 | 186.27 | 144 | 235 | 160 to 208 |  | 1 | 19 | 3 to 12 |
| 70 | 198.86 | 154 | 251 | 171 to 221 |  | 2 | 22 | 5 to 16 |
| 80 | 210.56 | 164 | 259 | 182 to 233 |  | 3 | 26 | 8 to 20 |
| 90 | 221.06 | 173 | 270 | 192 to 244 |  | 5 | 31 | 10 to 24 |
| 100 | 230.79 | 179 | 276 | 201 to 253 |  | 9 | 36 | 14 to 29 |

**Donor R-09**

**Sample Size = 500**

|  | **Number of Integration Sites** | | | |  | **Missed** | | |
| --- | --- | --- | --- | --- | --- | --- | --- | --- |
| **Top** | **Expect** | **Min** | **Max** | **95% CI** |  | **Min** | **Max** | **95% CI** |
| 10 | 39.557 | 18 | 62 | 28 to 51 |  | 0 | 4 | 0 to 2 |
| 20 | 56.55 | 31 | 82 | 42 to 70 |  | 0 | 9 | 0 to 5 |
| 30 | 68.88 | 41 | 95 | 53 to 83 |  | 0 | 15 | 2 to 9 |
| 40 | 78.29 | 48 | 109 | 62 to 93 |  | 1 | 19 | 5 to 14 |
| 50 | 86.17 | 55 | 121 | 69 to 101 |  | 5 | 25 | 8 to 20 |
| 60 | 93.14 | 60 | 131 | 75 to 109 |  | 8 | 32 | 13 to 26 |
| 70 | 99.43 | 67 | 138 | 81 to 115 |  | 12 | 37 | 17 to 31 |
| 80 | 105.28 | 72 | 144 | 86 to 122 |  | 16 | 46 | 22 to 38 |
| 90 | 110.53 | 75 | 150 | 91 to 127 |  | 22 | 53 | 28 to 44 |
| 100 | 115.39 | 77 | 152 | 95 to 132 |  | 27 | 61 | 33 to 51 |

The R-09 results seem to be a little more stable, though with a sample size of 1000, between 14 and 29 of the 100 largest clones could be missed. For a sample size of 500, between 33 and 51 of the largest 100 clones could be missed.

References

1. Brian T. Luke. Genetic algorithms and beyond. in Nature-inspired methods in chemometrics: genetic algorithms and artificial neural networks. (R Leardi, ed.) 1st ed. Amsterdam; Boston: Elsevier; 2003. Chapter 1.
